# Cellulose metabolism in halo(natrono)archaea: a comparative genomics study

**DOI:** 10.3389/fmicb.2023.1112247

**Published:** 2023-06-01

**Authors:** Alexander G. Elcheninov, Yaroslav A. Ugolkov, Ivan M. Elizarov, Alexandra A. Klyukina, Ilya V. Kublanov, Dimitry Y. Sorokin

**Affiliations:** ^1^Winogradsky Institute of Microbiology, Federal Research Centre of Biotechnology, Russian Academy of Sciences, Moscow, Russia; ^2^Department of Biotechnology, Delft University of Technology, Delft, Netherlands

**Keywords:** haloarchaea, cellulotrophic, genomics, CAZymes, cellulose, polysaccharides degradation

## Abstract

Extremely halophilic archaea are one of the principal microbial community components in hypersaline environments. The majority of cultivated haloarchaea are aerobic heterotrophs using peptides or simple sugars as carbon and energy sources. At the same time, a number of novel metabolic capacities of these extremophiles were discovered recently among which is a capability of growing on insoluble polysaccharides such as cellulose and chitin. Still, polysaccharidolytic strains are in minority among cultivated haloarchaea and their capacities of hydrolyzing recalcitrant polysaccharides are hardly investigated. This includes the mechanisms and enzymes involved in cellulose degradation, which are well studied for bacterial species, while almost unexplored in archaea and haloarchaea in particular. To fill this gap, a comparative genomic analysis of 155 cultivated representatives of halo(natrono)archaea, including seven cellulotrophic strains belonging to the genera *Natronobiforma, Natronolimnobius*, *Natrarchaeobius*, *Halosimplex*, *Halomicrobium* and *Halococcoides* was performed. The analysis revealed a number of cellulases, encoded in the genomes of cellulotrophic strains but also in several haloarchaea, for which the capacity to grow on cellulose was not shown. Surprisingly, the cellulases genes, especially of GH5, GH9 and GH12 families, were significantly overrepresented in the cellulotrophic haloarchaea genomes in comparison with other cellulotrophic archaea and even cellulotrophic bacteria. Besides cellulases, the genes for GH10 and GH51 families were also abundant in the genomes of cellulotrophic haloarchaea. These results allowed to propose the genomic patterns, determining the capability of haloarchaea to grow on cellulose. The patterns helped to predict cellulotrophic capacity for several halo(natrono)archaea, and for three of them it was experimentally confirmed. Further genomic search revealed that glucose and cellooligosaccharides import occurred by means of porters and ABC (ATP-binding cassette) transporters. Intracellular glucose oxidation occurred through glycolysis or the semi-phosphorylative Entner-Dudoroff pathway which occurrence was strain-specific. Comparative analysis of CAZymes toolbox and available cultivation-based information allowed proposing two possible strategies used by haloarchaea capable of growing on cellulose: so-called specialists are more effective in degradation of cellulose while generalists are more flexible in nutrient spectra. Besides CAZymes profiles the groups differed in genome sizes, as well as in variability of mechanisms of import and central metabolism of sugars.

## Introduction

Extremely halophilic archaea, belonging to the class *Halobacteria* (*Euryarchaeota* phylum), are abundant in natural terrestrial and deep-see hypersaline lakes, men-made solar salterns, rock salt deposits and saline soils. The well-studied majority of cultivated haloarchaea are growing aerobically on rich media containing peptides or simple sugars. Recently, however, a number of novel metabolic capacities of haloarchaea were discovered, including capability to grow anaerobically by sulfur respiration (Sorokin et al., 2016, 2017, 2021) or to grow aerobically with insoluble polysaccharides as the sole substrate (Sorokin et al., 2015, 2018, 2019a,b). Still, the haloarchaea bearing novel metabolic features are in total minority among cultivated representatives of this class with their unique metabolic machinery practically unexplored on biochemical or genomic level. Another question is whether the numerous haloarchaea growing on simple substrates may have any of the mentioned above properties, more specifically would the saccharolytic haloarchaea be capable of growth on insoluble polysaccharides.

Polysaccharides are degraded under the action of different types of enzymes, belonging to so-called carbohydrate-active enzymes (CAZymes, Drula et al., 2022). CAZymes included glycosidases (GHs), polysaccharide lyases (PLs), carbohydrate esterases (CEs), glycosyl transferases (GTs, mostly involved in carbohydrate biosynthesis) as well as auxiliary proteins (AAs) and proteins with carbohydrate binding domains (CBMs). Hydrolysis of exogenous insoluble polysaccharides demand extracellular CAZymes (in total majority – GHs), responsible for initial degradation steps occurred outside the cell. Currently, four model of CAZymes export and the consequent mechanisms of polysaccharides degradation in bacteria are suggested (Gardner and Schreier, 2021): (a) CAZymes are exported *via* outer-membrane vesicles (Elhenawy et al., 2014), (b) CAZymes are exported from periplasm *via* type II secretion system (Gardner and Keating, 2010), (c) cellulosome – CAZymes and specific carbohydrate-binding proteins are attached to the scaffoldins anchored to the cytoplasmic membrane (Artzi et al., 2017), and (d) S-layer-bound CAZymes and tapirins (specific binding protein) are attached to S-layer glycoproteins and pili (Conway et al., 2016; Lee et al., 2019). In turn, the mechanisms and enzymes, involved in polysaccharides hydrolysis in archaea and specifically in halophilic archaea are almost unknown. In particular, this is true for one of the most abundant polysaccharide on Earth – cellulose. Cellulose is a recalcitrant structural homopolysaccharide consisted of beta-1,4-linked D-glucose residues. Despite no variation in primary structure, different forms of celluloses distinguished from each other by degree of crystallinity and ratio and layout of crystalline and amorphous domain – so-called allomorphs (Uusi-Tarkka et al., 2021) which defines the variability of the enzymes involved in cellulose hydrolysis.

Extracellular cellulose hydrolysis resulted in formation of cellooligosaccharides (maximal – C6, Zhong et al., 2020), cellobiose and glucose. The last is less preferable since it is more accessible for competitors and accounts for the preference of cellulotrophic microorganisms to carry out the final steps of cellooligosaccharides hydrolysis intracellularly. Although, a few studies on cellooligosaccharides and cellobiose import in hyperthermophilic archaea *Pyrococcus furiosus* (Koning et al., 2001) and *Sulfolobus solfataricus* (Elferink et al., 2001) revealed that high-affinity ATP-binding cassette (ABC) transporters to be involved in this process, but nothing is known about haloarchaea. The mechanisms of glucose import into the cells as well as proteins involved in this process also are very poorly studied in haloarchaea. It seems the ABC transporters play a key role (Williams et al., 2019) in glucose import in halophilic archaea. Glucose is a single final product of cellulose hydrolysis, and its oxidation in haloarchaea occurred by means of (a) semi-phosphorylative Entner-Doudoroff pathway (Johnsen et al., 2001; Pickl et al., 2014), (b) modified glycolysis involving ketohexokinase and 1-phosphofructokinase (Altekar and Rangaswamy, 1991; Pickl et al., 2012) or (c) canonical glycolysis with ADP-dependent phosphofructokinase.

Recent developments in sequence technologies as well as the overall interest to haloarchaea resulted in a high number of available haloarchaeal genome sequences, both cultivated and uncultured (metagenome assembled genomes - MAGs). This is a good background for a comprehensive comparative genomic study to reveal the mechanisms of cellulose utilization in haloarchaea. The aim of this work was to use comparative genomics for comprehensive annotation of haloarchaeal CAZymes, involved in cellulose hydrolysis to reveal their cellulolytic machinery in the strains capable of growing on cellulose and to be able predicting this possibility for strains, for which it was not verified experimentally. Finally, basing on the sets of GHs and other CAZymes and auxiliary proteins we attempted to predict haloarchaea’s strategies of polysaccharides decomposing in hypersaline environments.

## Materials and methods

### Genome sequencing

Genomic DNA isolation, Illumina sequencing as well as genome assembly were performed as described earlier (Sorokin et al., 2018).

For strain AArcel5 additional sequencing using nanopore techonology (Oxford Nanopore Technology) was done. Genomic DNA of the strain was isolated using phenol-chloroform extraction (Gavrilov et al., 2016) and futher repurified using MagAttract HMW DNA Kit (Qiagen) according to manufacturer protocol. The DNA library was prepared with Rapid Barcoding Kit (SQK-RBK004, Oxford Nanopore Technologies). Sequencing was performed with FLO-MIN-106D flow cell (R9.4.1) and MinION device. Basecalling was performed using Guppy basecaller v.2.3.5 with flipflop model. In total of 94,910 reads were obtained by ONT sequencing (~152 Mbp). Assembly was performed as follows: Canu v.1.8 (Koren et al., 2017) was used to obtain *de novo* assembly using long ONT reads followed by Nanopolish v.0.11 (Loman et al., 2015) polishing with raw fast5 reads as well as several rounds of Pilon v.1.23 (Walker et al., 2014) polishing with Illumina reads.

### Genome and phylogenetic analyses

High quality genomes of cultivated haloarchaea were downloaded from IMG/M system (Chen et al., 2019); genomes, which were *de novo* sequenced during this work, were also previously annotated using IMG/M system. To exclude almost identical genomes AAI matrix was constructed using aai_matrix.sh (Rodriguez-R and Konstantinidis, 2016). Completeness levels of assemblies with >95% AAI with each other were estimated with CheckM v.1.1.5 (Parks et al., 2015): one assembly with better quality from each group will be selected for the further analysis.

For phylogenomic analysis based on the “ar122” set of conserved archaeal proteins, the sequences were identified and aligned in *in silico* proteomes of strains from AArcel and HArcel groups as well as described species within *Halobacteria* using the GTDB-tk v.1.2.0 with reference data v.89 (Chaumeil et al., 2019). The phylogenomic tree was constructed using RAxML v.8.2.12 (Stamatakis, 2014) with the PROTGAMMAILG model of amino acid substitution; local support values were 1,000 rapid bootstrap replications. Phylogenetic tree was visualized using iTOL v.6.5.2 (Letunic and Bork, 2019).

CAZymes genes were identified in the genomes using dbCAN v.2.0.11 (Zhang et al., 2018). Comparative analysis was performed with the complete set of revealed in each genome CAZymes as well as with families containing the enzymes with targeted (eg. endoglucanases) activities. Enzymes localization was predicted using SignalP v.6.0 (Teufel et al., 2022). Isoelectric points were estimated with IPC 2.0 (Kozlowski, 2021).

Putative carbohydrate-specific transporters as well as enzymes involved in central catabolism pathways were detected using blastp with characterized reference proteins, obtained from SwissProt (Boutet et al., 2016) and TCDB (Saier et al., 2014) databases, as queries and haloarchaeal genomes as subjects (e-value <10^−5^). Positive hits were manually checked with blast against SwissProt database. For ABC transporters only the gene clusters encoding at least substrate-binding protein and permease subunits were taken into account (ATPase was not since it is relatively nonspecific component).

Clusters of Orthologous Groups (COGs) were identified with IMG Pipeline (Chen et al., 2019). NMDS ordination was performed with vegan package.^1^

### Experimental support

Neutrophilic and alkaliphilic haloarchaea were cultivated on the medium prepared according to Sorokin et al. (2015). For the haloarchaea from salt lakes, a mineral base medium contained the following (g l^−1^): 240 NaCl, 5 KCl, 0.25 NH_4_Cl, 2.5 K_2_HPO_4_, pH 6.8. The medium was heat sterilized at 120°C for 30 min and after cooling supplemented with vitamin and trace metal mix (Pfennig and Lippert, 1966) (1 mL l^−1^ each) and 2 mM MgSO_4_. For alkaliphilic natronoarchaea from soda lakes, a sodium carbonate/bicarbonate buffered mineral base medium containing 4 M total Na^+^ included (g l^−1^): 190 Na_2_CO_3_, 30 NaHCO_3_, 16 NaCl, 5 KCl and 1 K_2_HPO_4_ with a final pH 10 after heat sterilization was supplemented with the same additions as the neutral base medium, except that the amount of Mg was two times lower and that 4 mM NH_4_Cl was added after sterilization. Finally, the ready to use alkaline base medium was mixed 1:3 with the neutral medium, resulting in the final pH of 9.6. Various forms of insoluble celluloses with different degrees of crystallinity were used as growth substrates at the final concentration of 1–2 g l^−1^.

## Results and discussion

### General genome properties and phylogenetic analysis

Genome properties of two cellulotrophic haloarchaeal groups, AArcel (alkaliphilic haloarchaea from soda lakes) and HArcel (neutrophilic haloarchaea from neutral salt lakes), were compared (Table 1). Two genome assemblies were obtained earlier [*Natronobiforma cellulositropha* AArcel2 (Sorokin et al., 2018) and *Halococcoides cellulosivorans* HArcel1 (Sorokin et al., 2019a)], one genome was resequenced and reassembled in the course of this work (*Natronobiforma cellulositropha* AArcel5^T^, see *Material and Methods* section) and the others (*Natronolimnobius* sp. AArcel1, *Natrarchaeobius* sp. AArcel7, *Halosimplex* sp. HArcel2 and *Halomicrobium* sp. HArcel3) were sequenced *de novo* in the course of this work. The G + C content of all the genome assemblies laid within rather narrow boundaries: 58.85–68.31%. In turn the genome sizes greatly varied from 2.72 Mbp (HArcel1) to 5.12 Mbp (AArcel7) leading to fairly broad range of a number of a protein-coding genes: 2641–4,769.

**Table 1:** General properties of genomes of haloarchaeal AArcel/HArcel strains.

Strain	IMG Genome ID	Genome size, bp	Gene count	Scaffold count	G + C, %	Reference
AArcel1	2,681,813,540	4,560,092	4,555	18	58.85	*de novo* sequencing
AArcel2	2,642,422,534	3,732,973	3,776	42	65.43	Sorokin et al., 2018
AArcel5	2,868,148,463	3,829,432	3,854	5	65.42	resequencing
AArcel7	2,808,606,451	5,121,137	4,820	22	62.82	*de novo* sequencing
HArcel1	2,681,813,541	2,723,120	2,757	1	65.74	Sorokin et al., 2019a
HArcel2	2,808,606,450	4,572,180	4,633	68	68.31	*de novo* sequencing
HArcel3	2,867,963,358	4,205,126	4,230	19	66.23	*de novo* sequencing

**Figure 1:**
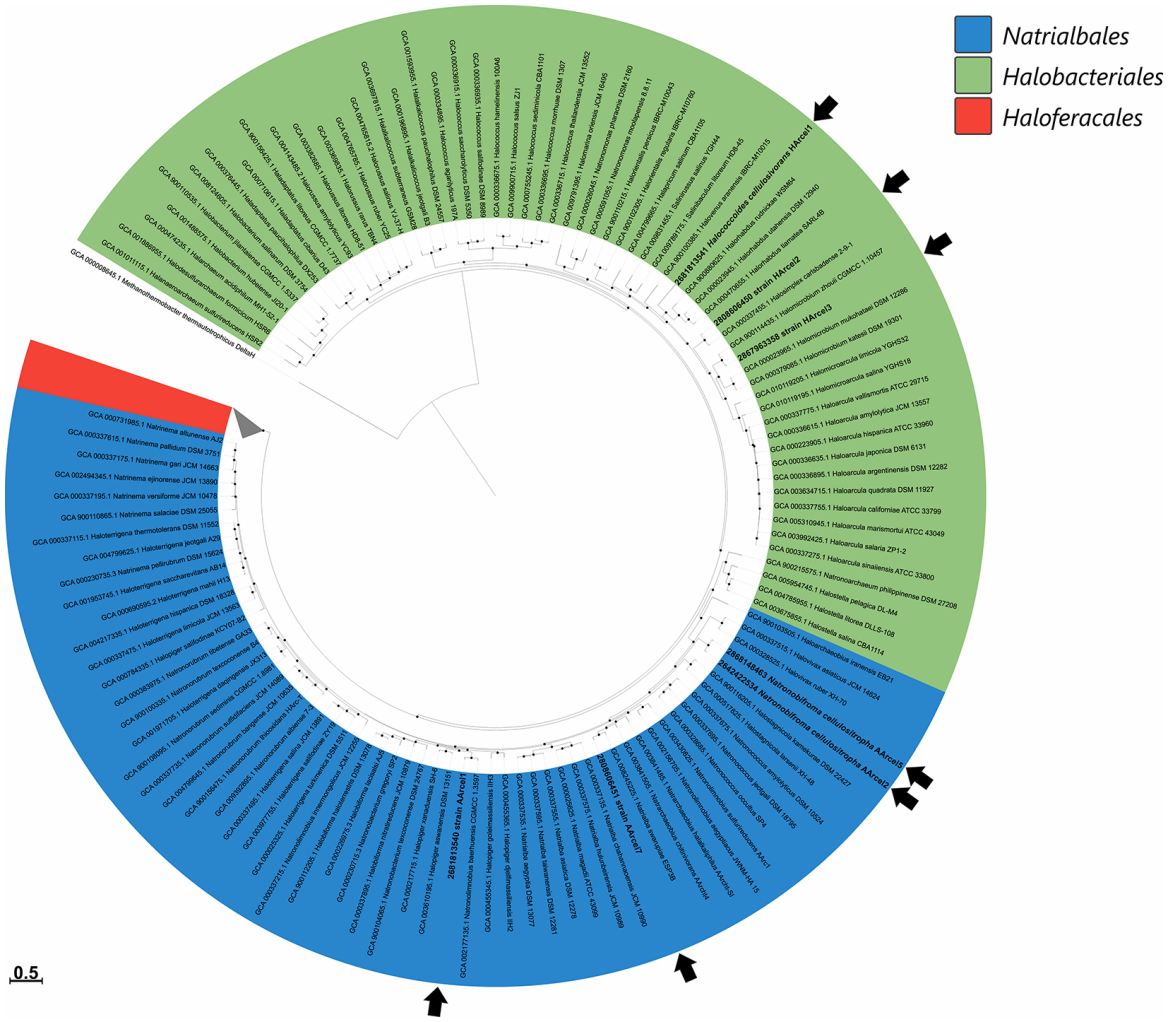
Maximum-likelihood phylogenetic tree of *Halobacteria* class based on 122 concatenated sequences of conservative archaeal proteins. Strains from AArcel/HArcel groups were marked by black arrows. The branch lengths correspond to the number of substitutions per site according to the corrections associated with the PROTGAMMAILG model in RAxML. The black circles at nodes indicate that the percentage of corresponding support values (1,000 rapid bootstrap replications) was above 50. *Methanothermobacter thermautotrophicus* DeltaH was used as an outgroup.

Currently all haloarchaea are affiliated with the class *Halobacteria* within the *Euryarchaeaota* phylum. Phylogenomic analysis of cellulotrophic strains based on “ar122” set of conserved proteins showed that natronoarchaeal AArcel strains belong to the order *Natrialbales*, while neutrophilic HArcel strains – to the order *Halobacteriales* (Figure 1) thus confirming the results of 16S rRNA gene and RpoB protein sequence-based phylogenetic analyses (Sorokin et al., 2018, 2019a). The seven cellulotrophic haloarchaea were relatively equally distributed on the haloarchaeal tree indicating polyphyletic origin of cellulotrophy in this class. To estimate occurence of this capability among haloarchaea 155 genomes of cultivated representatives of *Halobacteria* class, including 7 cellulotrophic strains were analyzed in respect to the presence of cellulose-active CAZymes.

### Cellulolytic capabilities of AArcel/HArcel strains compared with other haloarchaea

Currently there are 26 known CAZymes families (22 glycoside hydrolases and 4 polysaccharide monooxygenases) harboring the enzymes with confirmed cellulolytic activities *sensu lato* (including hydrolysis of cellooligosaccharides, e.g., beta-glucosidase or cellobiose phosphorylase, http://www.cazy.org; Supplementary Table S1): beta-glucosidase (GH1, GH2, GH3, GH30, GH39, GH116), endoglucanase (GH5, GH6, GH7, GH8, GH9, GH10, GH12, GH44, GH45, GH48, GH51, GH74, GH124, GH131, GH148), cellobiose/cellodextrin phosphorylase (GH94) and lytic cellulose monooxygenase (AA9, AA10, AA15, AA16). Among them, 13 families contain archaeal sequences and only 6 families contained biochemically characterized cellulases and related enzymes found in archaea (GH1, GH2, GH3, GH5, GH12 and GH116). Besides cellulases there is a number of auxiliary proteins responsible for binding and transportation of oligomers and glucose inside the cell.

To reveal the distribution of the cellulases *sensu lato* among the haloarchaeal genomes, the genes encoding selected GHs and AAs families members were searched in 155 high-quality genomes of representatives of *Halobacteria* class including 7 genomes of AArcel/HArcel strains, playing a role of positive controls as they are known to be cellulose-utilizing organisms (Sorokin et al., 2015; Supplementary Figure S1). The search showed that 117 of 155 genomes possess at least one gene encoding protein from the abovementioned families (11 families were found).

**Figure 2:**
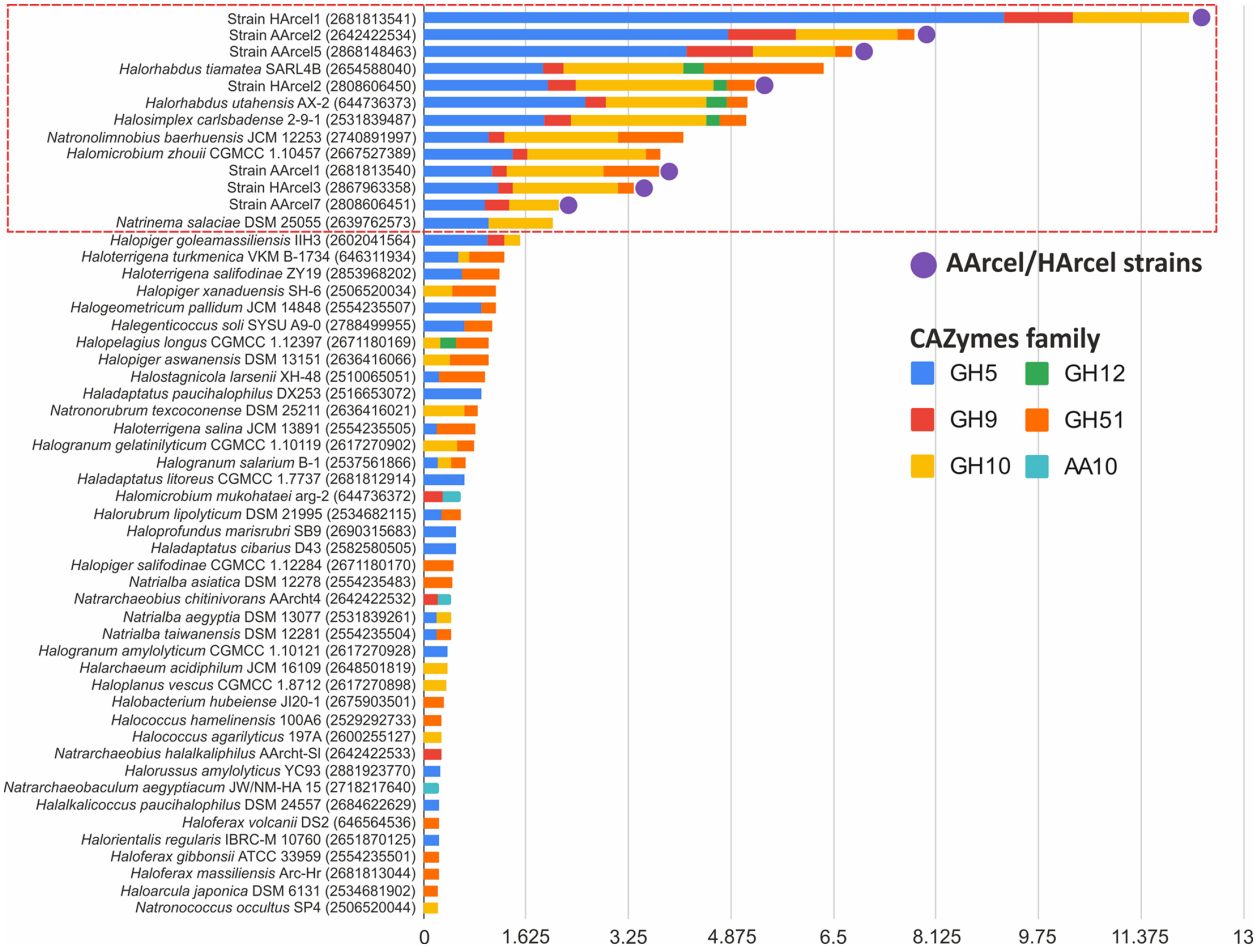
Relative abundance (gene number per1 Mbp) of putative endoglucanase genes found in 53 genomes of haloarchaea.

The cellulases genes were unequally distributed within these 117 genomes and the AArcel/HArcel strains with the confirmed ability to utilize native insoluble forms of cellulose (Sorokin et al., 2015) were among the top in the number of such genes per genome. Among other haloarchaea for which this capacity is yet unknown there were examples with high number of cellulase genes per genome as well as genomes encoded single or few cellulases and the transition from the first to the latter variaty was seamless. With such distribution, it appeared impossible to distinguish genuine cellulotrophic representatives using this cellulases *sensu lato* dataset which is, most probably, related to the fact that besides cellulases these GH families contain enzymes which only indirectly involved in cellulose decomposition. In this regard, a set of query CAZymes was limited to CAZymes families containing endoglucanases – enzymes, playing crucial role in cellulose depolymerization (Mandeep et al., 2021) and which can be considered as signature enzymes for cellulotrophic organisms. The search with the endoglucanases set (Figure 2) resulted in selection of a much narrower group of haloarchaeal genomes with a high probability to be capable of degrading cellulose, not only its smaller and soluble derivatives as cellobiose, cellooligosacharides or heteropolysaccharides, containing beta-1,4-glucose linkages in their backbone or side chains. In total, 13 strains were found capable of degrading cellulose including all AArcel/HArcel strains, for which an ability to grow on native celluloses was experimentally approved (Sorokin et al., 2015). Besides AArcel and HArcel strains, the following haloarchaea were predicted to be cellulotrophic: *Halosimplex carlsbadense* 2–9-1, *Halorhabdus tiamatea* SARL4B, *Halorhabdus utahensis* AX-2, *Halomicrobium zhouii* CGMCC 1.10457, *Natronolimnobius baerhuensis* JCM 12253 and *Natrinema salaciae* DSM 25055. Three of them, *N. baerhuensis* JCM 12253*, H. carlsbadense* 2–9-1 (JCM 11222) and *H. zhouii* CGMCC 1.10457 (JCM 17095), were acquired from the Japan Collection of Microorganisms (JCM, https://jcm.brc.riken.jp/en/) and their ability to grow on amorphous cellulose was confirmed in our laboratory, while the other three still need to be tested.

While inspecting the reference endoglucanase sets of these cellulotrophic strains, including both AArcel/HArcel with the confirmed growth on cellulose and *de novo* predicted cellulotrophs it became apparent that the true cellulotrophic archaea must possess multiple and variable GH5 and GH10 families glycosidases, as well as at least several representatives from the GH9 family [excluding *Natrinema salaciae* DSM 25055 (2639762573)]. It should be noted that characterized proteins from GH5 and GH9 are mainly endoglucanases, while the majority of GH10 glycosidases are endoxylanases (despite several endoglucanases are also known (Xue et al., 2015; Zhao et al., 2019) being the reason to include this family into the “endoglucanases” set). It is possible that the latter are indeed cellulases in haloarchaea or involved in hemicelluloses decomposition, which might contribute to a better availability of cellulose for cellulases.

**Figure 3:**
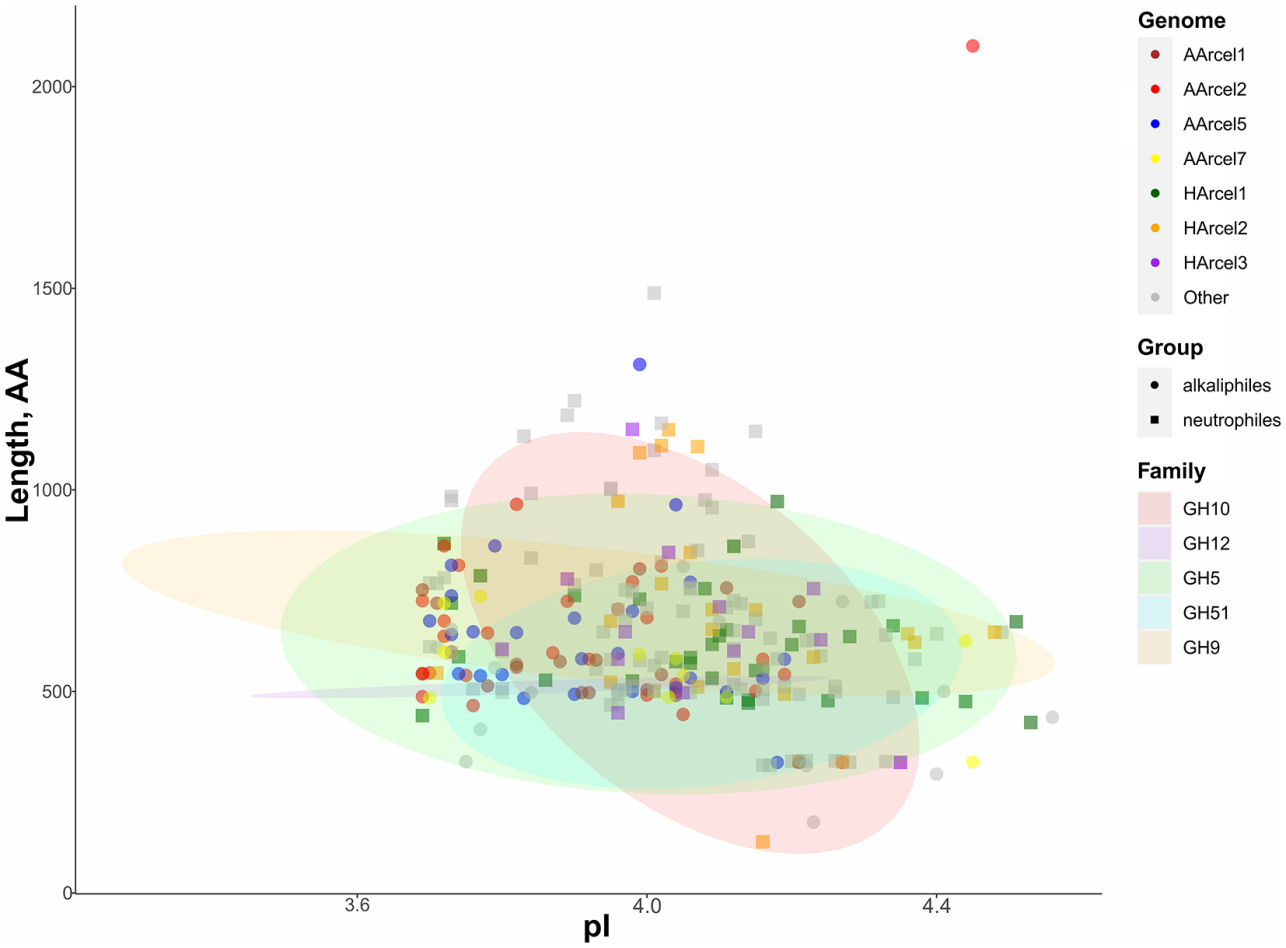
Characteristics of putative endoglucanases found in 13 genomes of cellulotrophic haloarchaea. Colors of the dots – genome assignment, shapes of the dots – assignment to neurtrophiles or alkaliphiles (based on literature data), ellipse color – enzyme family.

All putative endoglucanases encoded in the genomes of cellulotrophic haloarchaea were highly acidic having isoelectric point (pI) values from 3.86 to 4.56 (Figure 3; Supplementary Figure S2) which is linked with high salinity of their environments. Several alkaliphilic strains (AArcel1, AArcel2 and AArcel5) possessed slightly lower median pI values compared with neutrophilic haloarchaea, while AArcel7 and *Natronolimnobius baerhuensis* JCM 12253 had median pI values similar to neutrophiles indicating that environmental pH is not influencing the ratio of charged amino acids in these enzymes.

The genes encoding GH5 family glycosidases were the most numerous GH-encoding genes found in the genomes of 13 proven and predicted cellulotrophic haloarchaea. The number of GH5-encoding genes varied from 5 to 25 per genome. According to PFAM the length of a single GH5 catalytic domain is around 406 amino acids, while the lengths of the GH5-containing proteins in 13 cellulotrophic haloarchaea varied from 326 to 2,101 amino acids (Supplementary Figure S3). The data indicated that many of these proteins contained additional substrate-binding or other yet undetectable domains which can provide novel functionalities (Supplementary Table S2).

### Ecological strategies of cellulose-utilizing haloarchaea

**Figure 4:**
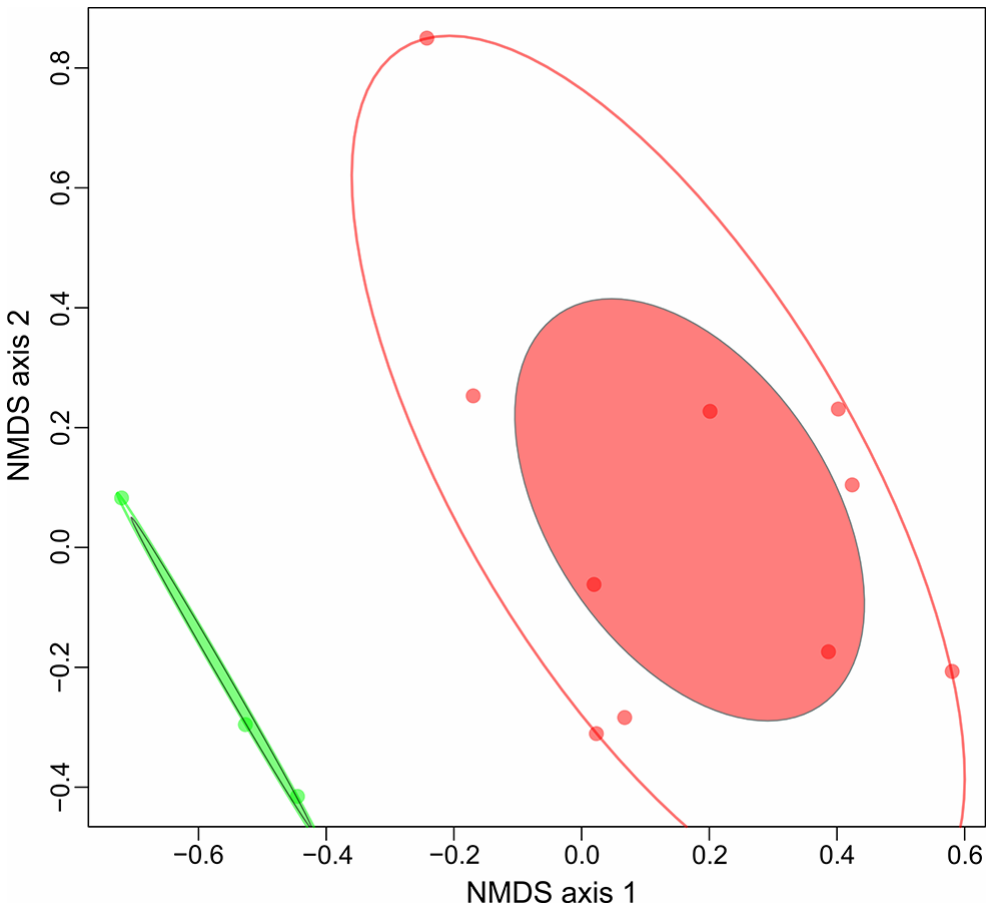
NMDS ordination plot (Bray, k = 2, stress value = 0.1263) of 13 haloarchaeal cellulolytic genomes based on CAZymes sets (with exception of GTs).

In our previous work on polysaccharidolytic haloarchaea (Sorokin et al., 2015) we proposed to divide all strains growing on cellulose into two groups: cellulotrophic and cellulolytic. The first are highly effective cellulose degraders, while the second are opportunists with broader substrate specificities, devouring many different oligo- and polysaccharides including the cellooligosaccharides released due to the action of the first group. In this respect, for 13 haloarchaea which either authoritatively or with high degree of probability being cellulotrophic an attempt has been made to reveal their lifestyle through the comparison of their CAZymes repertoire. Genome clustering of 13 genomes of cellulotrophic haloarchaea with NMDS ordinations was performed based on (i) a complete set of COGs found in the genomes and (ii) a set of CAZymes (excluding glycosyl transferases). Genome clusterization based on COGs gave no results since a relatively similar metabolism in terms of COGs functional categories was observed in all strains. Different results were obtained when CAZymes distribution among the genomes were used for clustering: two clearly separated groups comprised of (i) a compact cluster containing three strains (HArcel1, AArcel2 and AArcel5) and (ii) a larger and more diffused cluster comprising of other ten strains (Figure 4) were observed. We propose that the cellulose-utilizing microorganisms from the first group can be assigned to “specialists” while the second one contained “generalists.” Remarkably, the genomes of cellulotrophic specialists are smaller than the generalists: 2.7–3.8 Mb and 4.2–5.1, respectively (Table 1) supporting our assumptions on their behavior.

**Figure 5:**
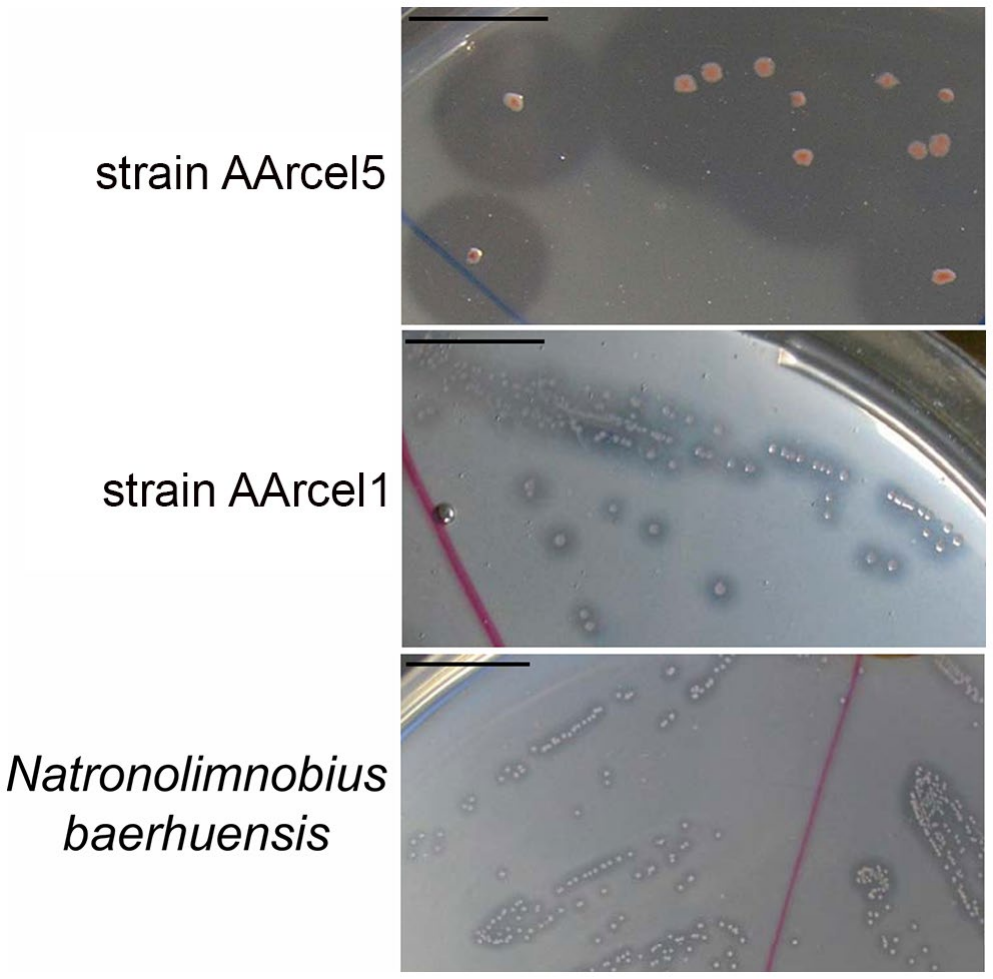
Amorphous cellulose hydrolysis by the colonies of strain AArcel5 (specialist), AArcel1 (generalist) and *N. baerhuensis* JCM 12253 (generalist). The bar scale is 1 cm.

Moreover, these two groups can be clearly distinguished not only by CAZymes repertoires and genome sizes but also by direct observation of ability to degrade cellulose. When growing on amorphous cellulose specialists form much larger hydrolysis zones in comparison with generalists (Figure 5).

**Figure 6:**
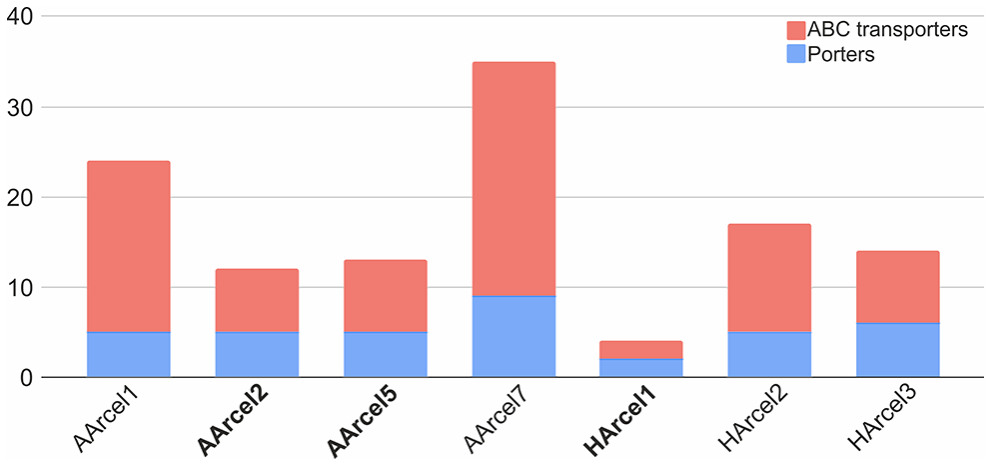
Number of putative transport system involved in carbohydrate transport AArcel/HArcel strains. Specialists are in bold.

Because of the action of numerous CAZymes cellulose is depolymerized to a single monomer, glucose. The question arose whether the central carbohydrate metabolism of cellulotrophic strains is uniform or the opposite is true. *In silico* reconstruction of the glucose/cellobiose/cellooligosaccharides import and glucose oxidation pathways in AArcel/HArcel strains with confirmed capability to grow on cellulose showed that glucose was transported into the cells by two different transport systems: (a) porters (superfamily 2.A according to TCDB) and ATP-binding cassette (ABC) transporters (superfamily 3.A.1 according to TCDB). Genes of phosphotransferase transport system (PTS) were absent in all genomes. In turn, cellooligosaccharides could be transported into the cells *via* ABC transporters as it was described for hyperthermophilic archaea (Koning et al., 2001). The number of genes encoding presumable carbohydrate transport systems components varied greatly between the genomes (Figure 6; Supplementary Table S3): HArcel1 possessed only 4 transporters (2 porters and 2 ABC transporters), while in the genome of AArcel7 35 transporters-encoding genes (9 porters and 26 ABC transporters) were found. A general observation is that the strains affiliated to specialists have less number of transporters than the generalists.

**Figure 7:**
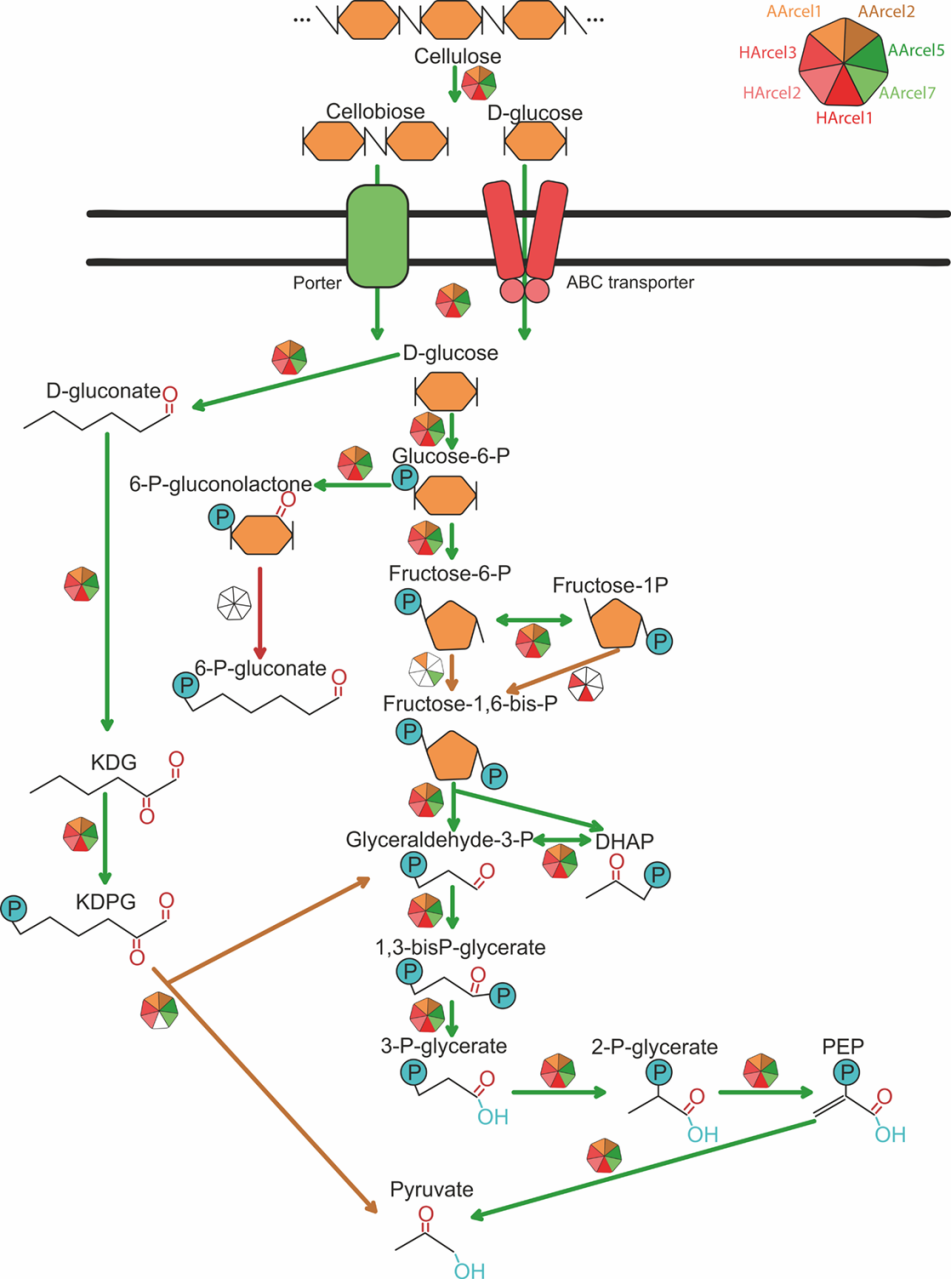
Glucose import and catabolism pathways found in AArcel/HArcel strains.

Genome analysis (Figure 7) revealed that glucose is metabolized *via* canonical-like glycolysis with ADP-phosphofructokinase (strains AArcel1 and AArcel7), haloarchaeal type of glycolysis (strains HArcel1 and HArcel3) or semi-phosphorylative Entner-Doudoroff pathway (all strains with exception of strain HArcel1).

Strain AArcel1 oxidizes glucose *via* glycolysis with ADP-phosphofructokinase as well as by complete semi-phosphorylative Entner-Doudoroff (KDPG) pathway. In the genomes of two closely related strains, AArcel5 and AArcel2, the genes encoding ADP-phosphofructokinase or 1-phosphofructokinase were absent indicating both glycolysis variants cannot be functional in this microorganism. Still, the genes of all KDPG pathway enzymes were found in the genomes of these haloarchaea. The glycolysis with ADP-phosphofructokinase as well as semi-phosphorylative KDPG pathway were predicted for AArcel7. Strain HArcel1 probably catabolized glucose only *via* glycolysis with phosphoglucomutase (performed the conversion of fructose-6-phosphate to fructose-1-phosphate, Lowry and Passonneau, 1969) and 1-phosphofructokinase and lacked KDPG pathway because KDPG aldolase gene was not found in the genome. Strain HArcel2 did not possess any variant of glycolysis due to the absence of the ADP-phosphofructokinase and 1-phosphofructokinase genes. Glucose was oxidized *via* KDPG-pathway in this microorganism. The genes encoding 1-phosphofructokinase and phosphoglucomutase were found in the genome of strain HArcel3 and thus it can utilize glucose *via* glycolysis like strain HArcel1. Complete semi-phosphorylative KDPG pathway was also predicted for this strain. Enzymes catalyzed common reactions for both the glycolysis and the KDPG pathway were present while glyceraldehyde-3-phosphate ferredoxin oxidoreductase (GAPOR), which often found in hyperthermophilic archaea, was absent in all studied strains. A gene of nonphosphorylating glyceraldehyde-3-phosphate dehydrogenase (GAPN) was found only in the AArcel7.

Summarizing the distribution of glucose oxidation pathways among the studied cellulotrophic haloarchaea it appears that specialists possessed only one glucose oxidation pathway, either glycolysis (HArcel1) or KDPG (AArcel2 and AArcel5). Generalists, in turn, possess two pathways (AArcel1, AArcel7 and HArcel3) with the only exception – HArcel2, oxidizing glucose *via* KDPG pathway. This seems to be associated with narrower metabolism of specialists. These results are in accordance with other findings, distinguished these two groups: specialists characterized by a narrow specialization on cellulose degradation, smaller genomes, larger repertoire of genes encoding putative endoglucanases and lower number and variety in sugar transporters. Generalists include less specialized strains with a much broader substrate spectrum, larger genomes encoding lower number of cellulases but higher number and variability of sugar transporters.

## Conclusion

The capacity of halophilic archaea to degrade various recalcitrant polysaccharides is of considerable interest for the understanding of their role in the mineralization of organic compounds in hypersaline environments and for search of extremely halo(alkali)stable extracellular CAZymes, attractive for the production of biofuel from lignocellulosic wastes since the pre-treatment step of this process is accomplished either with alkali or ionic liquids (Zavrel et al., 2009).

Large-scale analysis of all known CAZymes families containing cellulases encoded in the high-quality genomes of cultivated haloarchaea allowed to predict putative cellulotrophic strains. Since the dataset included the genomes of haloarchaea for which growth on and degradation of cellulose were experimentally confirmed and which therefore can be used as positive markers, these predictions allowed to propose a set of CAZymes-encoding genes indicative of the potential cellulotrophic lifestyle with a high degree of probability. Experimental validation of three out of seven cellulotrophic strains for which this property was not shown before confirmed their ability to grow on cellulose. The CAZymes patterns characteristic to cellulotrophic haloarchaea can serve as a tool for the comparative genomics-based identifying other haloarchaea carrying this trait.

Finally, genomic analysis followed by experimental verification of cellulase activity allowed dividing the cellulotrophic haloarchaea into two groups differed in strategies of cellulose utilization - specialists and generalists. The groups differed in efficiency of cellulose hydrolysis, CAZyme profiles, genome sizes, as well as in variability of mechanisms of import and central metabolism of sugars. Both groups are capable of growth on cellulose but specialists are more effective in cellulose degradation while generalists are more flexible to environmental changes, particularly to the changes in nutrient sources.

## Data availability statement

The datasets presented in this study can be found in online repositories. The names of the repository/repositories and accession number(s) can be found in the article/Supplementary material.

## Author contributions

AE, YU, and IK analyzed the genomes and run phylogenetic analysis. IE and AK were responsible for DNA isolation and genome sequencing libraries preparation. DS performed microbiological experiments. IK supervised the study. AE, IK, and DS wrote the manuscript. All authors contributed to the article and approved the submitted version.

## Funding

The work was supported by the Ministry of Science and Higher Education of the Russian Federation.

## Conflict of interest

The authors declare that the research was conducted in the absence of any commercial or financial relationships that could be construed as a potential conflict of interest.

## Publisher’s note

All claims expressed in this article are solely those of the authors and do not necessarily represent those of their affiliated organizations, or those of the publisher, the editors and the reviewers. Any product that may be evaluated in this article, or claim that may be made by its manufacturer, is not guaranteed or endorsed by the publisher.

## Footnotes

^1^https://CRAN.R-project.org/package=vegan
