# Supplementary material for: Cellulose metabolism in halo(natrono)archaea: a comparative genomics study
Source: Front Microbiol. 2023 Jun 1;14:1112247. doi: 10.3389/fmicb.2023.1112247 (PMC10267330; doi:10.3389/fmicb.2023.1112247)
Supplement: Supplementary file 4 [file Table_4.DOCX]

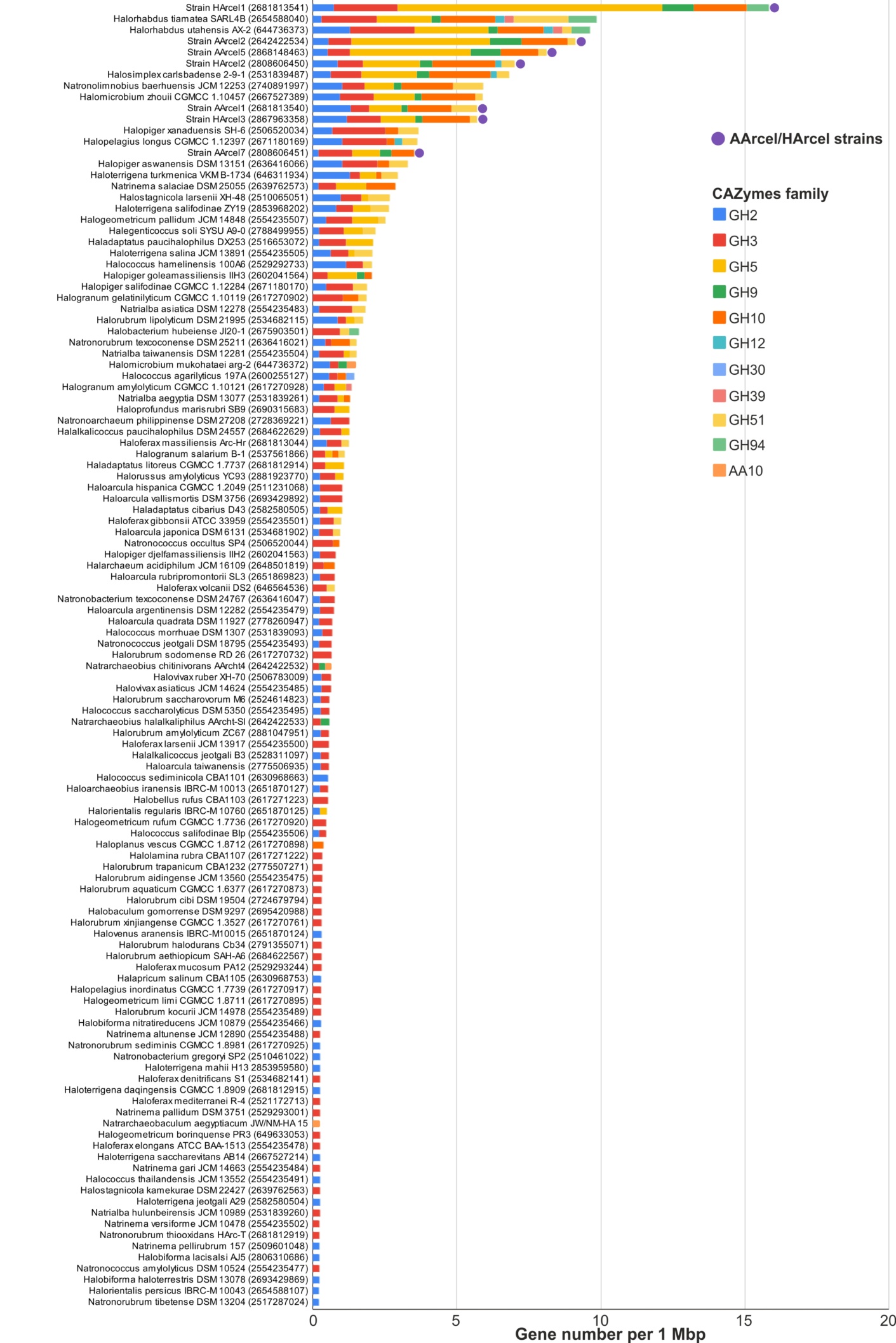


Supplementary Figure S1. Relative abundance (gene number per1 Mbp) of genes encoding enzymes involved in cellulose degradation found among 155 genomes of haloarchaea.


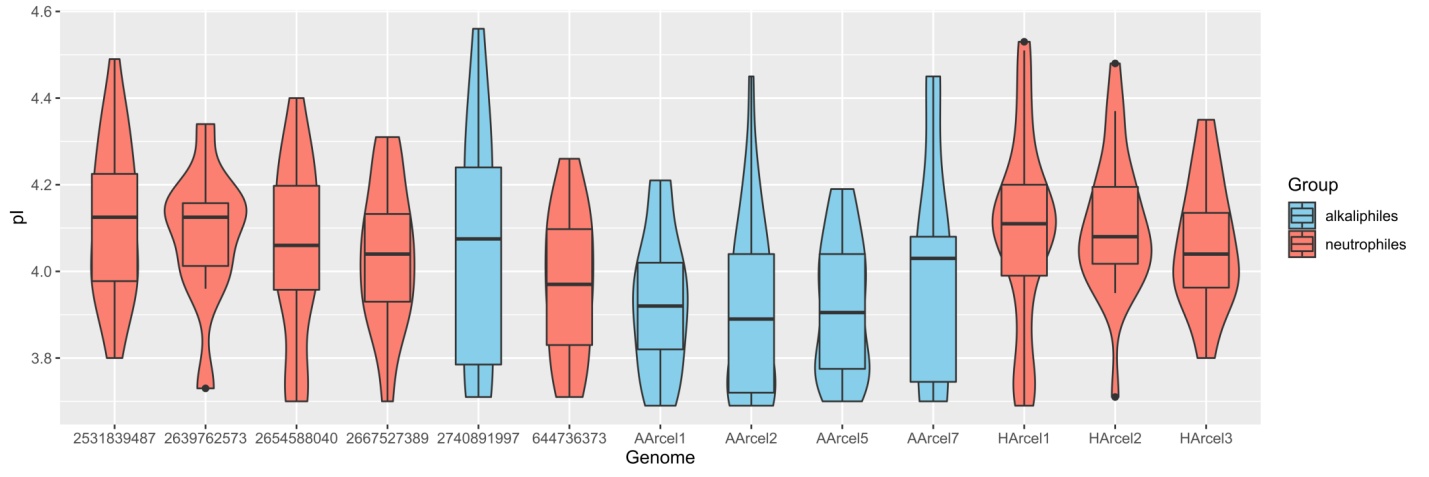


Supplementary Figure S2. Boxplot indicate distribution of isoelectric points of putative endoglucanases found in 13 genomes of cellulotrophic haloarchaea. Violin plot indicate the frequency of enzymes with respective pI Assignment to neutrophiles or alkaliphiles was based on information from the published descriptions of these strains.


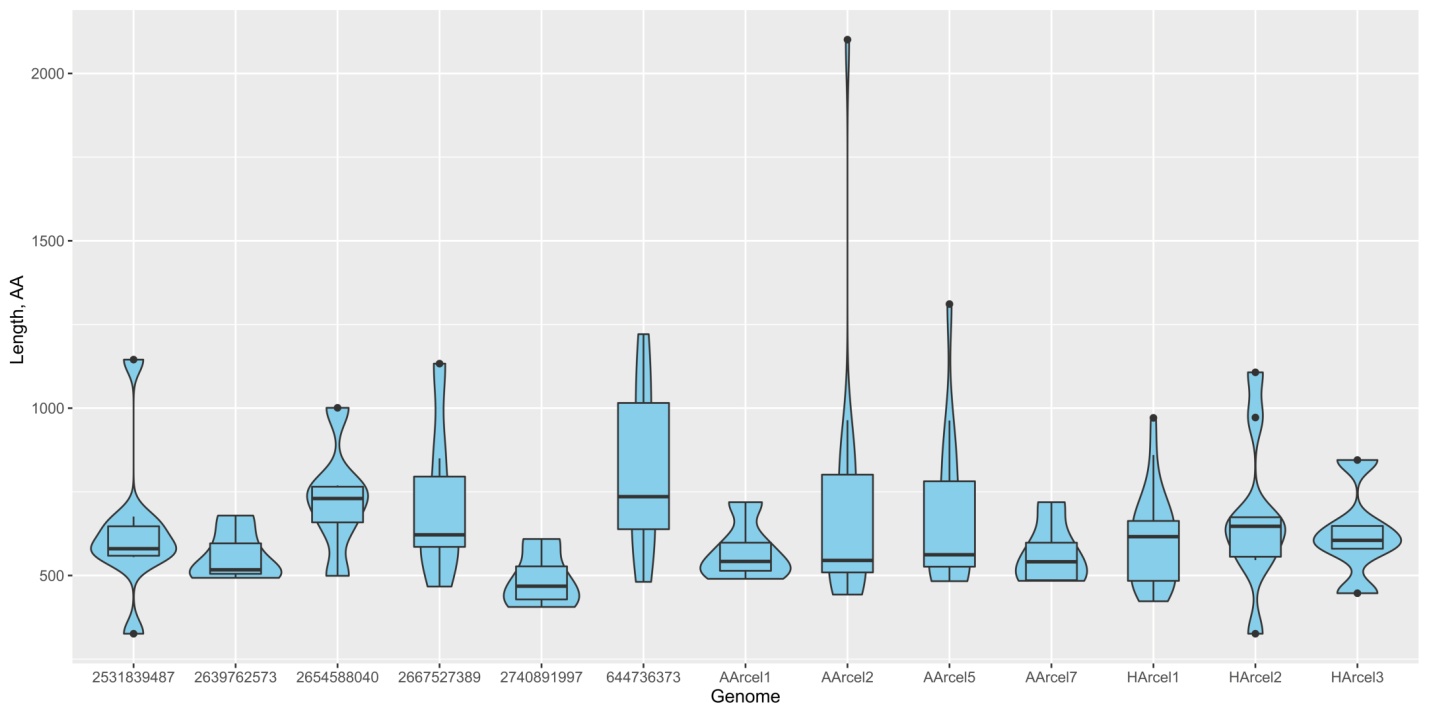


Supplementary Figure S3. Boxplot indicate distribution of lengths of GH5 glycosidases found in 13 cellulolytic haloarchaea. Violin plot indicate frequency of enzymes with following value.
